# Buspirone in obsessive-compulsive disorder: a potential dark horse?

**DOI:** 10.1192/bjo.2021.457

**Published:** 2021-06-18

**Authors:** Kabir Garg, Himanshu Tyagi

**Affiliations:** 1 Oxleas NHS Foundation Trust; 2 UCL Queen Square Institute of Neurology,The National Hospital for Neurology and Neurosurgery

## Abstract

**Aims:**

Pharmacological management of Obsessive-Compulsive Disorder (OCD) presents a challenge in modern psychiatry. While most patients respond preferably to serotonin re-uptake inhibitors (SRI), the response is usually delayed by several weeks leading to an insufficient short term management of anxiety. It is also frequently inadequate and needs higher doses and augmentation in many instances. Investigating newer pharmacological strategies to address such treatment gaps has always been of interest. Buspirone is a novel anxiolytic medication with additional weak antidepressant and poor anti-psychotic effects. It is the only medication in its category, i.e. azapriones. It has comparable anti-anxiety efficacy to that of benzodiazepines without their sedating or habit forming effects, and has been demonstrated to moderate serotonin and other monoamine neurotransmission with a favourable safety profile.

**Method:**

We reviewed the literature pertaining to the use of Buspirone in OCD for both as a primary anti-obsessive agent and for a potential secondary role in management of chronic anxiety and/or anxiety disorders comorbid to OCD.

**Result:**

The results of a number of case reports and open trials have been positive while controlled trials have shown contradictory results. In a double blind RCT comparing clomipramine and buspirone, significant improvement was found in both groups with no differences between the two. Further two trials observing buspirone augmentation of clomipramine and fluoxetine treatment respectively, in a double-blind placebo controlled design reported significant improvement in the treatment as opposed to the placebo arm. Another double-blind placebo controlled study of buspirone augmentation of fluvoxamine resistant patients did not show significant benefits as an anti-obsessional agent, but notable anxiolytic effects were reported. In all the trials buspirone was largely well tolerated and did not pose any significant interactions with other psychotropic agents or dependence potential.

**Conclusion:**

Buspirone is a pharmacologically unique agent with a good safety profile. Given the robust anxiolytic effects of this Peron along with complex neurotransmission modulatory effects coupled with a favourable tolerance and dependents profile might make buspirone an attractive novel pharmacological agent for augmentation in OCD . Further controlled studies to better establish effectiveness and deciphering if certain patients may respond to its use over others, are warranted

